# The role of femoral obliquity angle and T1 pelvic angle in predicting quality of life after spinal surgery in adult spinal deformities

**DOI:** 10.1186/s12891-021-04823-3

**Published:** 2021-11-30

**Authors:** Andrea Perna, Luca Proietti, Amarildo Smakaj, Calogero Velluto, Maria Concetta Meluzio, Giuseppe Rovere, Daniela Florio, Gianfranco Zirio, Francesco Ciro Tamburrelli

**Affiliations:** 1 Fondazione Policlinico Universitario Agostino Gemelli – IRCCS, Largo A. Gemelli, 8, 00168 Rome, Italy; 2 Istituto di Ortopedia e Traumatologia, Università Cattolica del Sacro Cuore, Rome, Italy

**Keywords:** Adult spinal deformities, Sagittal imbalance, Femoral obliquity angle, Spinopelvic parameters, Spinal deformity correction

## Abstract

**Background:**

Adult spinal deformities (ASD) represent a growing clinical condition related to chronic pain, disability and reduction in quality of life (QoL). A strong correlation among spinal alignment, spinopelvic parameters and QoL after spinal fusion surgery in ASD patients was thoroughly investigated over the last decade, However, only few studies focused on the relationship between lumbo-pelvic-femoral parameters - such as Femoral Obliquity Angle (FOA), T1 Pelvic Angle (TPA) and QoL.

**Methods:**

Radiological and clinical data from 43 patients surgically treated with thoracolumbar posterior spinal fusion for ASD between 2015 and 2018 were retrospectively analyzed. The primary outcomes were the correlation between preoperative spino-pelvic-femoral parameters and postoperative clinical, functional outcomes and QoL. Secondary outcomes were: changes in sagittal radiographic parameters spino-pelvic-femoral, clinical and functional outcomes and the rate of complications after surgery.

**Results:**

Using Spearman’s rank correlation coefficients, spinopelvic femoral parameters (FOA, TPA, pre and post-operative) are directly statistically correlated to the quality of life (ODI, SRS-22, pre and post-operative; > 0,6 strong correlation, *p* <  0.05). Stratifying the patients according pre preoperative FOA value (High FOA ≥ 10 and Normal/Low FOA <  10), those belonging to the first group showed worse clinical (VAS: 5.2 +/− 1.4 vs 2.9 +/− 0.8) and functional outcomes (ODI: 35.6+/− 6.8 vs 23.2 +/− 6.5) after 2 years of follow-up and a greater number of mechanical complications (57.9% vs 8.3% *p* <  0.0021).

**Conclusion:**

Based on our results, preoperative FOA and TPA could be important prognostic parameters for predicting disability and quality of life after spinal surgery in ASD patients and early indicators of possible spinal sagittal malalignment. FOA and TPA, like other and better known spinopelvic parameters, should always be considered when planning corrective surgery in ASD patients.

## Background

Nowadays adult spinal deformities (ASD) represent a growing clinical condition related to chronic pain, disability and reduction in quality of life (QoL) [1, 2] . ASD are often associated with spine aging due to the intervertebral disc degeneration, paravertebral muscles weakening and bone quality reduction [3]. The first sign of spinal degeneration observed clinically and radiographically is reduction of lumbar lordosis (LL) which is generally compensated by pelvic retroversion (PR) and hips and knees flexion [4, 5]. PR could cause reduction of anterior acetabular continence thus altering bilateral hip range of motion (ROM), favoring secondary hips osteoarthritis and raising the risk of prosthetic dislocation in total hip replacement patients [6, 7].

Major spine surgery with fixation of the lumbar spine and lumbar-sacral junction is often necessary for severe ASD correction [8]. Several surgical options are available for the correction of spinal deformities in the sagittal and coronal planes, such as open posterior surgery with multiple Posterior Column Osteotomies (PCO) or Pedicle Subtraction Osteotomies (PSO), Transforaminal Lumbar Interbody Fusion (TLIF), Minimally Invasive Surgery (MIS) combined with eXtreme Lateral Interbody Fusion (XLIF) or Anterior Lumbar Interbody Fusion (ALIF) [2, 9–11].

A strong correlation between spinal alignment, spinopelvic parameters - Pelvic Index (PI), Pelvic Tilt (PT), Sacral Slope (SS), Sagittal Vertical Axis (SVA) - and QoL after spinal fusion surgery in ASD patients has been reported [12]. However, only few studies focused on the relationship between lumbo-pelvic-femoral parameters - such as Femoral Obliquity Angle (FOA),T1 Pelvic Angle (TPA) and QoL [3].

The purpose of the current study was to investigate the relationship between spinopelvic and lumbopelvic-femoral radiologic parameters in ASD patients treated surgically with posterior thoracolumbar spinal fusion and the impact of these parameters on the QoL.

## Methods

### Study setting and design

The present investigation is an Institutional Review Board-approved retrospective analysis of surgically treated patients with long thoracolumbar posterior spinal fusion for ASD at our institution (single-surgical team) between 2015 and 2018. All patients included in the current study were clinically and radiographically evaluated 1, 3, 6, 12 months after surgery and annually thereafter. All procedures performed were in accordance with the 1964 Helsinki declaration and their further amendments. A written informed consent for scientific purposes and clinical data collection was obtained according to institutional protocol.

### Participants and eligibility criteria

All patients affected by ASD that underwent spinal corrective surgery at our institution between December 2015 and November 2018 were potentially eligible for the study.

Inclusion criteria were: (I) a complete clinical and radiological data set; (II) a minimum follow-up of 24 months.

Exclusion criteria were: (I) Preoperative bone density - studied by Dual Energy X-Ray Absorptiometry (DEXA) with t-score < − 2.0 (measured on the femoral neck); (II) Neoplastic diseases; (III) Rheumatic diseases with ossification of the posterior longitudinal ligament (e.g. ankylosing spondylitis); (IV) Spinal infections;; (IV) Previous surgery for total hip arthroplasty.

### Variables

The primary outcomes were the correlation between preoperative spino-pelvic-femoral parameters and postoperative clinical, functional outcomes and QoL (based on the ODI score and the SRS-22). Secondary outcomes were: changes in sagittal radiographic parameters spino-pelvic-femoral, clinical and functional outcomes and the rate of complications after surgery.

### Radiological outcomes

**Fig. 1:**
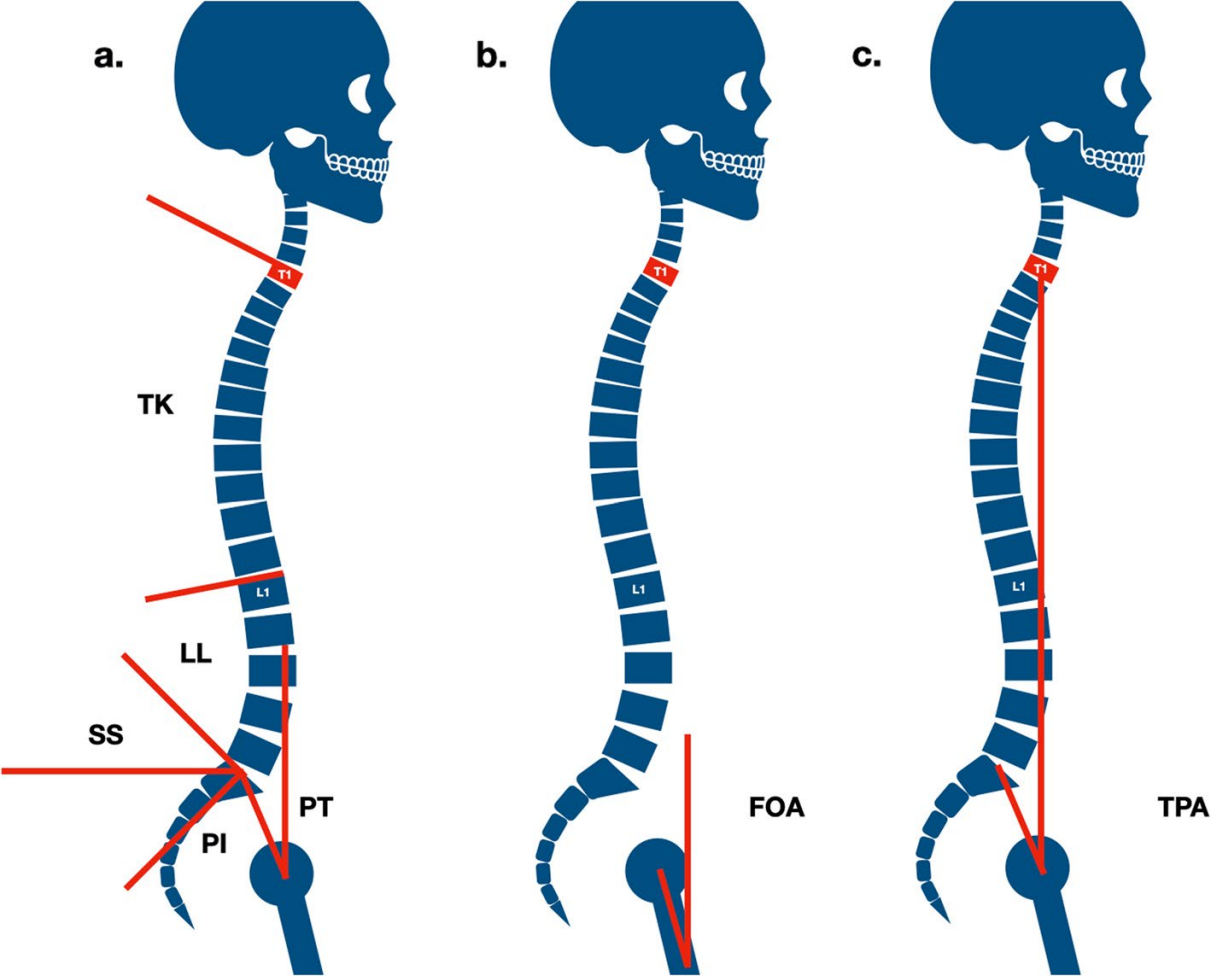
**a**. Standard spino-pelvic parameters; **b**. Femoral Obliquity Angle; **c**. T1 Pelvic Angle

Analyzed data were collected from the Institutional Picture Archiving and Communication system (PACS). Antero-posterior (AP) and Lateral full-length spine X-Ray in standing position performed preoperatively, immediately postoperatively (during the first week after surgery, when the patient was able to assume the orthostatic position) and 12 and 24 months postoperatively were retrieved and reviewed, using a dedicated workstation (Advantage Windows Workstation; GE Medical Systems, Milwaukee USA). The following parameters were measured in all examined X-Ray: PI, PT, SS, LL (from L1 to S1), Thoracic Kyphosis (TK, from T1 to T12), SVA, FOA, TPA, Coronal Cobb (CC) of major thoracolumbar/lumbar curve. FOA represents the angle between the femoral axis and the vertical. TPA is a measurement technique influenced by the spinal sagittal balance and the pelvic retroversion which seems to be strictly related to clinical outcomes [13]. TPA was calculated as the angle between a line connecting the midpoint of the femoral heads to the midpoint of the sacral endplate and a line connecting the midpoint of the femoral heads to the center of T1 [13] (Fig. 1). Radiographic measurements were independently performed by three authors: two senior spinal surgeons (F.C.T., L. P.) and one orthopedic resident (A.P.).

### Clinical and functional evaluation

Clinical evaluations was performed preoperatively, 6, 12 and 24 months after surgery, using a ten-points itemized Visual Analogue Scale (VAS) for lumbar (VAS-l) and radicular (VAS-r) pain, the Oswestry Disability Index (ODI) score and the Scoliosis Research Society Outcomes Questionnaire (SRS-22).

### Statistical analysis

Data were reported as means and standard deviations (SD). The achieved results were analyzed by using the χ2 test for the Oswestry Disability Index. Mann Whitney’s test was used to analyze the results of the VAS and spino-pelvic-femoral parameters variations. The inter-rater reliability (IRR) between the three evaluators was calculated using a Fleiss’ kappa statistic. Spearman’s rank correlation coefficient was used to evaluate if spino-pelvic-femoral parameters had a significant correlation with QoL (ODI and SRS-22 scores). The analysis of the sample normality performed with Shapiro e Wilk test demonstrated a non-normal distribution hence it was not indicated to perform the analysis of variances with the ANOVA test. Statistical significance was established for a *p*-value < 0.05.

## Results

### Participants

Forty-three patients (32F, 11 M) were enrolled in the current study. Patients data are summarized in Table 1.

**Table 1:** Demographic and surgical data of enrolled patients

	Mean (+/−) SD	Percentage (%)
**N of patients**	43	
**Age**	61.1 (+/− 5.8)	
**Sex**	32 F; 11 M	F/M ratio: 2.9
**BMI**	27.8 (+/− 7.3)	
**Previous Spine Surgery**	7	16.3%
**Surgical Technique**		
Hybrid MIS	9	21%
Posterior Open	34	79%
**Upper and Lower IV**		
*D2- S1*	3	7%
*D4-S1*	10	23.2%
*D10-S1*	30	69.8%
**Iliac Fixation**	32	74.2%
**Screw Implanted**	904	
**Cage Implanted**	74	
TLIF	48	64.9%
XLIF	24	32.4%
ALIF	2	2.7%
**Mean Surgical Time**	376.6 (+/− 83.8)	
**Mean Blood Loss**	395.5 (+/− 42.4)	
**Complications**	24	55.7%
*Biomechanic*	13	54.2%
*Infection*	2	8.3%
*Others*	9	37.5%
**Follow up (months)**	37.4 (+/−12.6)	

*ALIF* Anterior Lumbar Interbody Fusion, *BMI* Body Mass Index, *IV* Instrumented Vertebra, *MIS* Minimally Invasive Surgery, *SD* Standard Deviation, *TLIF* Transforaminal Lumbar Interbody Fusion, *XLIF* eXtreme lateral Lumbar Interbody Fusion. In “Others” falls: thromboembolism (4 cases), abdominal wall twitching (2 cases), abnormal surgical wound (5 cases)

### Surgical data

All patients were treated by a single surgical team. Special attention was paid to hip extension during patient positioning on the operative table. Electrophysiologic monitoring systems were used during surgical procedures. There were no intraoperative complications recorded, excluding 2 cases of dural tear at lumbar level repaired by direct suture with non-absorbable stitches and fibrin glue. Posterior open surgery was performed in 34 (79%) patients, while hybrid MIS surgery (Minimally Invasive lateral or anterior approach combined with open posterior surgery) in 9 (21%) patients.

Overall 904 trans pedicle screws and 74 interbody cages were implanted. Postoperative complications were recorded in 24 patients (55.7%). Concerning biomechanical complication were documented 5 cases of Proximal Junctional Kyphosis (PJK), 4 cases of Rod Fractures (RF), and 4 cases of Screw Loosening (SL).

**Fig. 2:**
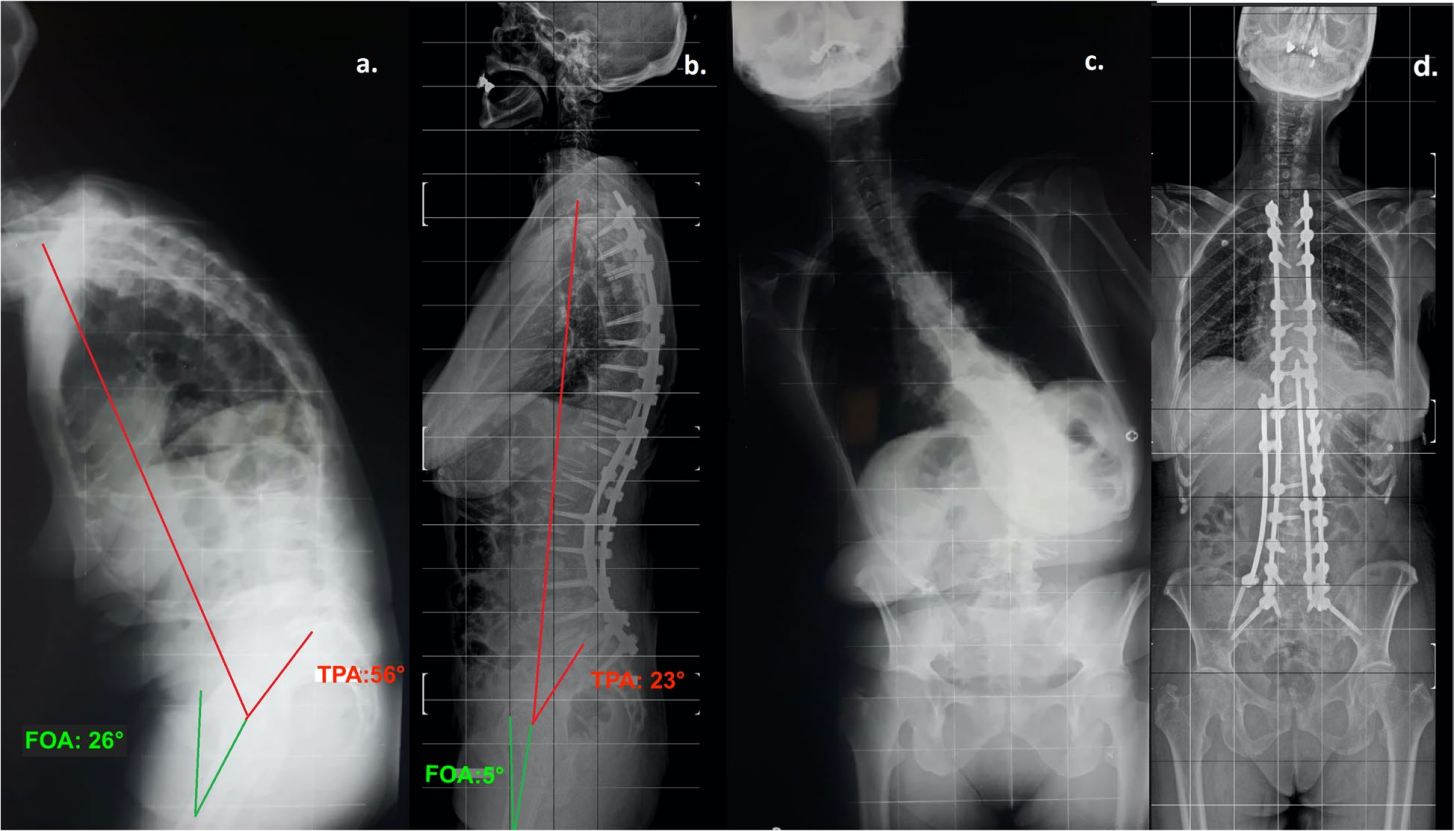
Example of a patients belonging to Group A (High FOA, > 10°); preoperative (**a**, **c**) and 24 months follow up (**b**, **d**) full spine standing radiographs of a 71 years-old female patient showing the correction of sagittal and coronal balance, FOA and TPA reduction after surgery and no instrumentation failure

**Fig. 3:**
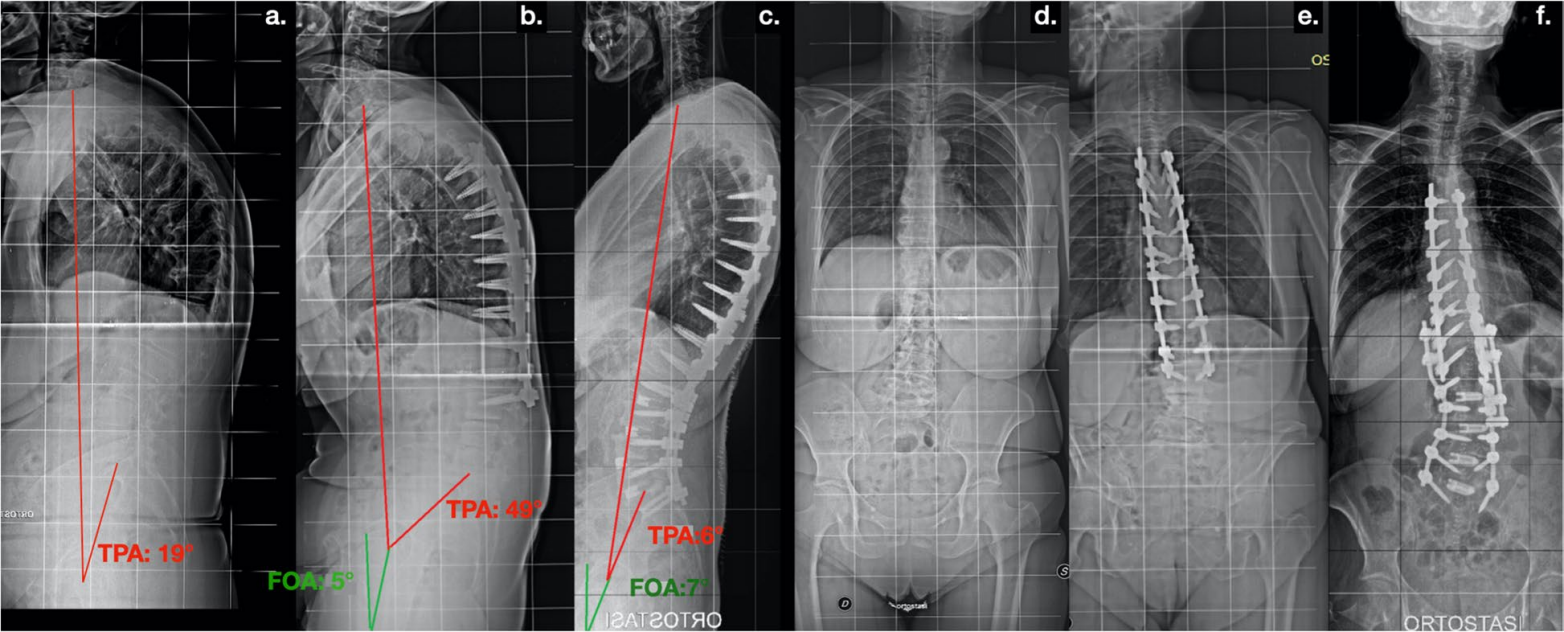
Patient belonging to Group A (Normal/Low FOA, < 10°); **a** d full spine standing radiographs of a 67 years-old female patient suffering from thoracic hyper kyphosis; **b**, **e** postoperative full spine standing radiographs 7 years after surgical hyper kyphosis correction performed in another hospital that show an increase of TPA. **c**, **f** The last postoperative radiographs show: the correction of sagittal imbalance, the restoration of lumbar lordosis, and reduction of FOA and TPA

Other data were summarized in Table 1. Examples of surgery performed were reported in Figs. 2 and 3.

### Radiological findings

The interobserver reliability was calculated by determining Fleiss’ kappa (0.799, 95% CI: 0.734–0.871) for the 3 raters. The radiographic data changed between preoperative value to 24 months follow up values as follows: SVA from 91.7 (+/− 18.2) mm preoperative to 42.1 (+/− 15.1) mm (*p* = 0.001), FOA from 12.9 (+/− 1.9)° to 7.8 (+/− 1.1)° (*p* = 0.014) and TPA from 30.5 (+/− 6.9)° to 22.3 (+/− 7.4)° (*p* < 0.001). No significant statistical variation was observed between 12 months follow up and 24 follow up measurement therefore radiographic data at 12 months of follow up were not included in our analysis.

A strong direct correlation was found respectively between preoperative FOA/preoperative SVA and preoperative TPA/preoperative SVA (respectively: r = 0.714, *p* = 0.001; r = 0.771, *p* < 0.001) using the Spearman’s rank correlation coefficient. Radiological findings were resumed in Table 2.

**Table 2:** Sagittal alignment data of the patients before surgery and 24 months after surgery

	Pre Operative	Post Operative (24 months of FU)	***P*** value
**Parameters**			
SVA (mm)	91.7 (+/− 18.2)	42.1 (+/− 15.1)	0.001
PI	45.6 (+/− 5.7)	44.3 (+/− 6.1)	> 0.05
PT	27.4 (+/− 4.1)	20.3 (+/−7.1)	0.027
SS	18.1 (+/−3.7)	25.2 (+/−4.3)	0.039
LL	28.7 (+/− 7.2)	41.6 (+/− 5.4)	< 0.001
PI-LL mismatch	17.1 (+/− 5.0)	4.0 (+/− 3.5)	< 0.001
TK	31.0 (+/−8.9)	31.9 (+/− 8.1)	> 0.05
FOA	12.9 (+/−1.9)	7.8 (+/−1.1)	0.014
TPA	30.5 (+/− 6.9)	22.3 (+/−7.4)	< 0.001
Major coronal curve	31.8 (+/−6.4)	14.5 (+/−3.1)	0.003
**Clinical Outcomes**			
VAS lumbar	8.1 (+/− 1.1)	3.9 (+/− 1.4)	0.022
VAS radicular	7.5 (+/−1.0)	3.4 (+/− 1.2)	0.005
ODI (%)	49.1 (+/−7.8)	28.2 (+/− 6.3)	0.044
SRS 22	3.1 (+/− 0.6)	2.0 (+/− 0.7)	0.021

*FOA* Femoral Obliquity Angle, *LL* Lumbar Lordosis, *PI* Pelvic Incidence, *PI-LL* PI minus LL, *PT* Pelvic Tilt, *ODI* Oswestry Disability Index, *SRS-22* Scoliosis Research Society questionnaire, *SS* Sacral Slope, *SVA* Sagittal Vertical Axis, *TPA* T1 Pelvic Angle, *VAS* Visual Analogue Scale

### Clinical and functional outcomes

The VAS-l improved from a pre-operative score of 8.1 (+/− 1.1) to a value of 3.9 (+/− 1.4) (*p* = 0.022) at the 24 months evaluation. The VAS-r improved from a preoperative score of 7.5 (+/− 1.0) to a value of 3.4 (+/− 1.2) (*p* = 0.005) after 24 months of follow-up. The ODI improved from a preoperative score of 49.1 (+/− 7.8) to a 24-months postoperative score of 28.2 (+/− 6.3) (*p* = 0.044). The SRS-22 improved from a preoperative score of 3.1 (+/− 0.6) to a 24-months postoperative score of 2.0. (+/− 0.7) (*p* = 0.021). No significant statistical variation was observed between 12 months follow-up and 24 follow-up measurement. Clinical and functional results were resumed in Table 2.

### Correlations between QoL (ODI,SRS-22) and spino-pelvic-femoral parameters (FOA, TPA)

Using Spearman’s rank correlation coefficient, a strong direct correlation between preoperative FOA, TPA and preoperative/postoperative ODI, SRS-22 was found. A strong direct correlation was also observed between postoperative FOA, TPA value, ODI and SRS-22 (Table 3). Thus as these two angles increase, there is an increase in disability, and a reduction in QoL.

**Table 3:** Relationship among QoL (ODI, SRS-22) and radiographic spino-pelvic-femoral (FOA, TPA) parameters. (Spearman’s Regression coefficient, *p*-value)

Parameters	ODI pre	ODI post (24 m)	SRS 22 pre	SRS 22 post (24 m)
FOA pre	0.633	0.760	0.701	0.766
	*p* = 0.0024	*p* = 0.0231	*p* = 0.0032	*p* = 0.0021
FOA post (24 m)	0.647	0.737	0.689	0.755
	*p* = 0.0013	*p* = 0.0039	*p* = 0.0091	*p* = 0.0074
TPA pre	0.640	0.765	0.759	0.719
	p = 0.005	p = 0.0032	*p* = 0.0014	p = 0.0021
TPA post (24 m)	0.454	0.632	0.681	0.560
	*p* = 0.0017	*p* = 0.0062	*p* = 0.0044	*p* = 0.0037

*FOA* Femoral Obliquity Angle, *ODI* Oswestry Disability Index, *SRS 22* Scoliosis Research Society 22 questionnaire, *TPA* T1 Pelvic Angle

### Subgroup analysis

Stratifying the patients according pre preoperative FOA value, 2 groups were identified: (A) High FOA (≥ 10) and (B) Normal/Low FOA (< 10). Patients belonging to group A showed worse clinical and functional outcomes after 2 years of follow-up compared to those of group B and a greater number of mechanical complications - such as Proximal Junctional Kyphosis (PJK), Rod Fractures (RF), Screw Loosening (SL) (57.9% vs 8.3% *p* < 0.0021) as summarized in Table 4.

**Table 4:** Patients features after division of patients in to two groups (A High FOA, B Normal/Low FOA)

**Group A (High FOA)** ***n*** **= 19,**			
	***Mean (+/− SD)***		
**Age**	65.3 (+/−5.1)		
**Sex**	F:16, M:3		
	*Pre-Operative*	*Post-Operative (24 months of FU)*	*P value*
**FOA**	12.2 (+/−0.9)	9.5 (+/−1.7)	0.002
**PTA**	36.7 (+/− 4.6)	22.7 (+/− 3.2)	0.0054
**ODI (%)**	56.4 (+/− 7.2)	35.6 (+/− 6.8)	0.0024
**SRS 22**	3.6 (+/− 0.5)	2.5 (+/− 0.8)	< 0.05
**VAS l**	8.6 (+/− 1.1)	5.2 (+/− 1.4)	0.007
**VAS r**	7.2 (+/− 1.0)	4.1 (+/−1.6)	0.005
**Biomechanics complications**		11 (57.9%)	
*SL*	3 (27.2%)		
*PJK*	4 (36.4%)		
*RF*	4 (36.4%)		
**Revision Surgery rate**	7 (36.8%)		
**Group B (Normal/Low FOA)** ***n*** **= 24**			
	***Mean (+/− SD)***		
**Age**	66.7 (+/− 6.3)		
**Sex**	F:18, M: 6		
	*Pre-Operative*	*Post-Operative (24 months of FU)*	*P value*
**FOA**	8.2 (+/− 0.7)	6.3 (+/− 1.1)	0.023
**PTA**	25.5 (+/−4.3)	18.4 (+/− 2.2)	0.0001
**ODI (%)**	43.2 (+/− 4.5)	23.2 (+/− 6.5)	0.0049
**SRS 22**	2.7 (+/− 0.4)	1.5 (+/− 0.3)	0.0003
**VAS l**	7.5 (+/−1.1)	2.9 (+/− 0.8)	> 0.0001
**VAS r**	7.3 (+/− 1.1)	2.8 (+/−0.9)	> 0.0001
**Biomechanics complications**		2 (8.3%)	
*SL*	1 (50%)		
*PJK*	1 (50%)		
*RF*	_		
**Revision Surgery rate**	1 (4.1%)		

*FOA* Femoral Obliquity Angle, *FU* Follow Up, *ODI* Oswestry Disability Index, *SRS-22* Scoliosis Research Society questionnaire, *PJK* Proximal Junctional Kyphosis, *RF* Rod Fractures, *SD* Standard Deviation, *SL* Screws Loosening, *SS* Sacral Slope, *SVA* Sagittal Vertical Axis, *TPA* T1 Pelvic Angle, *VAS* Visual Analogue Scale

## Discussion

### Main findings

In the current series we observed a significant improvement of clinical and functional outcomes between preoperative and after 24 months of follow-up evaluation, considering all patients enrolled. Among the global sagittal radiographic parameters, preoperative FOA and TPA had a significant correlation with both ODI and SRS-22 (preoperative and postoperative). A strong correlation was also found between postoperative FOA, TPA value and both postoperative ODI and SRS-22.

When patients were divided according to preoperative FOA measurement, those with a preoperative FOA greater than 10° (Group A) had a higher rate of biomechanical complications and revision surgery. Patients belonging to group A show worse clinical and functional results (VAS-l, VAS-r, ODI, SRS-22) with respect to patients belonging to group B.

Our results are in accordance with the current knowledges, and they strengthen the correlation between sagittal alignment and the lower limb compensatory mechanisms.

**Fig. 4:**
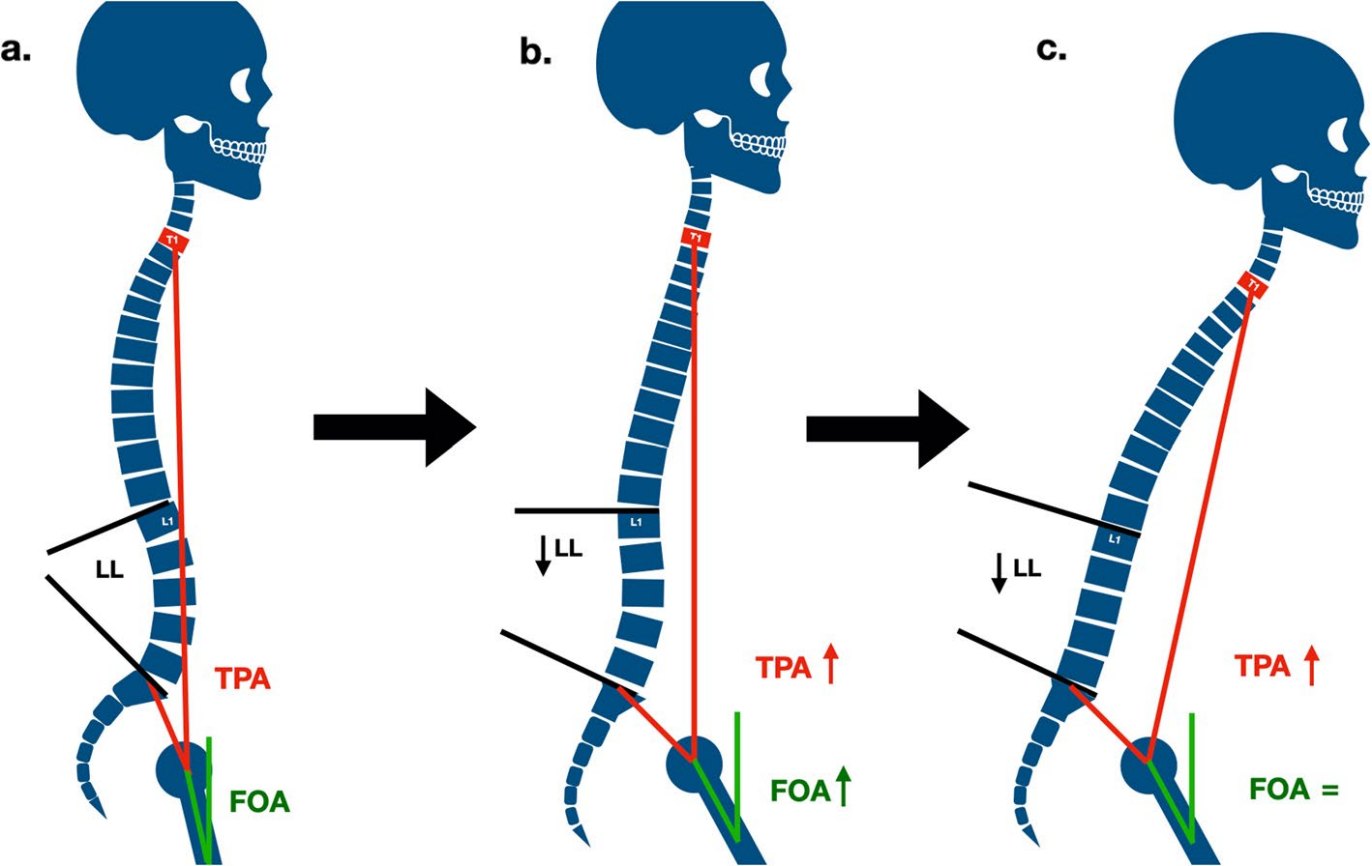
Mechanism of sagittal malalignment modified from Roussouly et al. classification [15] for FOA e TPA variation. **a** Physiological sagittal alignment; **b** Compensation phase: reduction of lumbar lordosis (LL) compensated by pelvic retroversion (PR) and hips flexion, increasing TPA and FOA, no trunk inclination; **c** Decompensation phase: pelvic retroversion and further increase of TPA and anterior trunk inclination

Spinal sagittal malalignment evolution was described by Roussouly et al. [14]. They identified three phases known as: (I) normal, (II) compensation and (III) decompensation. During the compensation phase, no increase in SVA but an increase in PT was observed. When compensation mechanisms were overcome, the decompensation phase began with SVA increase and hip flexion (Fig. 4).

Many authors in the Literature demonstrated that the sagittal spinal balance is the most correlated parameter with clinical outcomes and disability, such as ODI and SRS-22 [1, 16]. Moreover, patients with sagittal malalignment appear to have more frequent disability, chronic pain, and worse clinical outcomes than patients with coronal plane imbalances, both pre- and postoperatively [1].

In fact, spinal and spinopelvic parameters like LL, TK, PI, PT, SS, SVA, and their correlation with clinical outcomes have been extensively studied by spinal surgeons over the past decade [14, 17–19]. However, only a few studies nowadays examine spino-pelvic-femoral parameters such as FOA and TPA.

TPA is an important global measure of sagittal spinal deformity: it is the sum of SVA and PT which respectively represent measures of trunk and pelvic postural compensation mechanisms during ASD [20, 21].

FOA, also known as proximal femoral angle (PFA), is the angle between the femoral axis and the vertical, calculated using the mean value of the right and left femur [3, 15, 22]. When the pelvis exhausts its compensatory functions to maintain an *“economic”* sagittal balance, it is necessary for the femurs to move forward, increasing pelvic retroversion and FOA [14]. In particular, when there is a hip flexion FOA increases whereas it decreases during hip extension. Clinical relevance of FOA has been partially examined in pediatric patients affected by spondylolisthesis revealing worse QoL when FOA increases [23, 24]. For this reason, FOA should be considered a global parameter of low extremities CMs during ASD as it is the result of hip and knee flexion.

Patient surgical positioning is crucial in the correction of ASD. Corrective surgery is generally performed in a prone position which has great impact on spinal sagittal alignment. In particular, Benfanti et al. [25] demonstrated that prone positioning of the patient in maximal hip extension causes an increase and preservation of lumbar lordosis that is essential during ASD corrective surgery. In fact, during hip extension there is a pelvic anteversion and consequently decreasing of PT and TPA. FOA decreases too because the femoral axis becomes more parallel to the vertical. Yasuda et al. [26] investigated the impact of positioning on sagittal alignment in patients with ASD suggesting that LL in supine position radiographs is approximately equal to LL in the prone position. This evidence should be helpful in surgical ASD planning.

Skeletal spino-pelvic and low extremities postural changes are not the only CMs which occur during ASD. The role of the paravertebral and psoas major muscles in maintaining the sagittal balance is not negligible. Therefore, preoperative hip surgical planning in patients with ASD should keep into consideration paravertebral and psoas major muscles.

### Clinical implications

As shown by our data, the FOA and TPA are strictly connected with the SVA, and an increase in these parameters could be predictive of a global sagittal malalignment. TPA is a parameter that the spine surgeon cannot neglect when choosing the Upper Instrumented Vertebra (UIV) during corrective surgery planning whereas FOA should be taken into consideration by hip surgeons too, especially during proximal femoral osteotomy [27, 28].

Based on our results, preoperative FOA and TPA could be important prognostic parameters for predicting disability and quality of life after spinal surgery in ASD patients and early indicators of possible spinal sagittal malalignment.

### Limitations

The current study had some limitations. In fact, the retrospectively collected data, the relatively small patient number and the absence of any control group could affect the present investigation level of evidence. Therefore, further comparison studies with larger case series and longer follow-up are necessary to strengthen our data.

## Conclusion

A strong correlation is present between FOA, TPA and functional clinical outcomes associated with QoL. ASD patients with FOA >  10 ° and an increased TPA appear to have worse clinical and functional outcomes both pre and post-operative after 2 years of follow-up.

FOA and TPA, like other and better known spinopelvic parameters, should always be considered when planning corrective surgery in ASD patients.

## Data Availability

The datasets used and analyzed during the current study are available from the corresponding author on reasonable request.

## Abbreviations

ALIF: Anterior Lumbar Interbody Fusion
AP: Antero-posterior
ASD: Adult Spinal Deformity
BMI: Body Mass Index
CC: Coronal Cobb
CMs: Compensatory mechanisms
DEXA: Dual Energy X-Ray Absorptiometry
FGP: Fondazione Policlinico Gemelli
FOA: Femoral Obliquity Angle
IV: Instrumented Vertebra
LL: Lumbar Lordosis
MIS: Minimally Invasive Surgery
ODI: Oswestry Disability Index
PACS: Picture archiving and communication system
PCO: Posterior Column Osteotomies
PFA: Proximal femoral angle
PI: Pelvic index
PJK: Proximal Junctional Kyphosis
PR: Pelvic retroversion
PSO: Pedicle Subtraction Osteotomies
PT: Pelvic Tilt
QoL: Quality of Life
RF: Rod fractures
ROM: Range of motion
SD: Standard Deviation
SL: Screw loosening
SRS-22: Scoliosis Research Society Questionnaire
SS: Sacral Slope
SVA: Sagittal Vertical Axis
TK: Thoracic Kyphosis
TLIF: Transforaminal Lumbar Interbody Fusion
TPA: T1 Pelvic Angle
XLIF: eXtreme Lateral Interbody Fusion
VAS: Visual Analogue Scale
